# Deep reinforcement learning enables adaptive-image augmentation for automated optical inspection of plant rust

**DOI:** 10.3389/fpls.2023.1142957

**Published:** 2023-07-07

**Authors:** Shiyong Wang, Asad Khan, Ying Lin, Zhuo Jiang, Hao Tang, Suliman Yousef Alomar, Muhammad Sanaullah, Uzair Aslam Bhatti

**Affiliations:** ^1^School of Mechanical and Automotive Engineering, South China University of Technology, Guangzhou, China; ^2^Metaverse Research Institute, School of Computer Science and Cyber Engineering, Guangzhou University, Guangzhou, China; ^3^College of Food Science, South China Agricultural University, Guangzhou, China; ^4^School of Information and Communication Engineering, Hainan University, Haikou, China; ^5^Zoology Department, College of Science, King Saud University, Riyadh, Saudi Arabia; ^6^Department of Computer Science, Bahauddin Zakariya University, Multan, Pakistan

**Keywords:** adaptive image augmentation, deep reinforcement learning, deep Q-learning, automated optical inspection, semantic segmentation

## Abstract

This study proposes an adaptive image augmentation scheme using deep reinforcement learning (DRL) to improve the performance of a deep learning-based automated optical inspection system. The study addresses the challenge of inconsistency in the performance of single image augmentation methods. It introduces a DRL algorithm, DQN, to select the most suitable augmentation method for each image. The proposed approach extracts geometric and pixel indicators to form states, and uses DeepLab-v3+ model to verify the augmented images and generate rewards. Image augmentation methods are treated as actions, and the DQN algorithm selects the best methods based on the images and segmentation model. The study demonstrates that the proposed framework outperforms any single image augmentation method and achieves better segmentation performance than other semantic segmentation models. The framework has practical implications for developing more accurate and robust automated optical inspection systems, critical for ensuring product quality in various industries. Future research can explore the generalizability and scalability of the proposed framework to other domains and applications. The code for this application is uploaded at https://github.com/lynnkobe/Adaptive-Image-Augmentation.git.

## 1 Introduction

Automated optical inspection (AOI) provides a flexible and efficient method of object monitoring. In agriculture, AOI can be used for early screening of leaf diseases to support timely intervention to prevent leaf rust. Leaf rust is a type of plant disease also known as red spot disease or sheep beard. There are 4,000 known species of leaf rust that attack a wide range of crops such as beans, tomatoes, and roses (Liu et al., 2022; Bhatti et al., 2023). Disease spots first appear as white and slightly raised spots on the lower cuticles of the lower (older) leaves of mature plants. Over time, the disease spots become covered in reddish-orange spore masses. Later, pustules form and turn yellow-green and eventually black. Severe infestations can cause foliage to chlorosis, deform, and eventually fall off (Jain et al., 2019; Bhatti et al., 2021; Lu et al., 2023; Wang et al., 2023; Yang et al., 2022; Zhang et al., 2022). The spread of this disease will seriously affect agricultural production and cause huge losses. Thus, detecting plant disease and rust is very important and effective for protecting plant growth and development, improving crop yield and quality, reducing pesticide use, and saving time and cost (Bhatti et al., 2022; Shoaib et al., 2023).

Artificial intelligence-enhanced AOI methods based on computer vision and deep learning are promising solutions for the adaptive identification of plant diseases (Liu and Wang, 2021). Algorithms that incorporate the two major computer vision tasks—classification and detection—have been widely used in plant disease detection. In terms of classification algorithms, Sethy et al. (2020) used convolutional neural networks (CNNs), ResNet50, to extract features, which were then fed to a support vector machine (SVM) for the disease classification, achieving an F1 score of 0.9838. Zhong and Zhao (2020) proposed three methods based on the DenseNet-121 deep convolutional network: regression, multi-label classification, and focal loss function to identify apple leaf diseases and improve the detection accuracy in unbalanced plant disease datasets. In terms of detection algorithms, Zhou et al. (2019) proposed a fast rice disease detection method based on the fusion of FCM-KM and Faster R-CNN to improve detection accuracy and reduce detection time. Sun et al. (2020) proposed a CNN-based multi-scale feature fusion instance detection method based on the improved SSD to detect corn leaf blight on complex backgrounds, with the highest average precision reaching 91.83%.

The classification and detection of plant diseases are only possible to judge whether the disease occurs in certain locations (Di and Li, 2022; Khan et al., 2022; Yan et al., 2022; Deng et al., 2023; Wang et al., 2023). Using computer vision segmentation algorithms, the size and shape of plant rust spots can be obtained (Wang et al., 2021; Ban et al., 2022; Shoaib et al., 2022; Zhang et al., 2022; Dang et al., 2023; Wang et al., 2023), and the severity of rust occurrence can be quantitatively evaluated. He et al. (2021) proposed an asymmetric shuffle convolutional neural network (ASNet) based on Mask R-CNN to segment three diseases, including apple rust, with an average segmentation accuracy of 94.7%. Lin et al. (2019) proposed a U-net-based CNN to segment powdery mildew from cucumber leaf images at the pixel level. Unfortunately, compared with the classification and detection of diseases, there is still little research on applying deep learning segmentation networks for rust identification.

In the study of rust detection, the size of the available data set is limited, and manual labeling requires a lot of time and effort. The traditional solution to image augmentation is to perform simple image processing, which has been verified to improve the performance of plant image segmentation. Lin et al. (2019) proposed improving the U-net segmentation network by using image augmentation technology to expand the training set to train the semantic segmentation model better. Zhang et al. (2022) proposed the DMCNN model, which obtained twice the data after image augmentation and achieved an average apple disease detection rate of more than 99.5%. The research proves that sample size and data quality are critical to improving detection accuracy. Unfortunately, whether there is redundancy in the data set obtained by image augmentation or whether the data quality is good or bad (Elmore and Lee, 2021; Dang et al., 2023; Xiong et al., 2023) is a question worth exploring. Blind pursuit of a sample size for inappropriate image augmentation may adversely affect the model.

Several image augmentation methods have been proposed, such as rotation and cropping. However, no single approach can always outperform others, and the image quality generated by these augmentation methods is uncertain. In other words, the bottleneck of current image augmentation methods is that it is difficult to define the optimal augmentation operation to achieve the most significant performance improvement for semantic segmentation. Currently, multiple augmentation methods are generally used together: all methods for the complete image set, one for a separated subset, or one for a randomly sampled subset. However, none of these assignment mechanisms can guarantee the best match between an image and an available augmentation method. To overcome this problem, deep reinforcement learning (DRL)-based image augmentation methods have been proposed (Yang et al., 2023). DRL is a machine learning technique that enables a software agent to optimize its decision-making policy by interacting with its environment (Zhou et al., 2021). Le et al. (2022) stated that DRL can automatically learn how to augment datasets effectively. Qin et al. (2020) developed a novel automatic learning-based image augmentation method for medical image segmentation, using DRL to model the augmentation task as a trial-and-error process.

However, image augmentation and image segmentation were previously trained in separate ways (Di and Li, 2022). The image segmentation results cannot provide feedback to the DRL-based image augmentation model. Therefore, we propose a DRL-enabled adaptive image augmentation framework based on the Deep Q-learning (DQN) algorithm and the semantic segmentation model, DeepLab-v3+, for apple rust detection. DQN learns the Q-value function with a deep neural network and uses the experience playback and the target network to improve the stability and learning effect (Xu et al., 2022). The main contributions of this study are as follows:

(1) A DRL-enabled adaptive image augmentation framework is proposed to adaptively select the best-matched image augmentation methods according to the image features. This way, an effective augmented image set is constructed from the original image set.
(2) The DeepLab-v3+ model is applied. It is pre-trained by the original image set and retrained in conjunction with the augmentation image set. The model is retrained in a transfer-learning way, featuring fast fine-tuning. The retrained model outputs average performance over the test image set as an evaluation index for the augmented image. Furthermore, the evaluation index provided feedback to the DRL model as a reward.
(3) The superiority of the DRL-enabled adaptive image augmentation framework is verified by comparing it with other image augmentation methods and semantic segmentation models over a set of performance indexes.
(4) The main finding is that the DRL-enabled adaptive image augmentation framework can best match image augmentation methods with the image features and the underlying segmentation model.

This paper provides an end-to-end, robust, and effective method for segmenting rust spots at the pixel level, providing a valuable tool for farmers and botanists to assess the severity of rust.

## 2 Method

**Figure 1:**
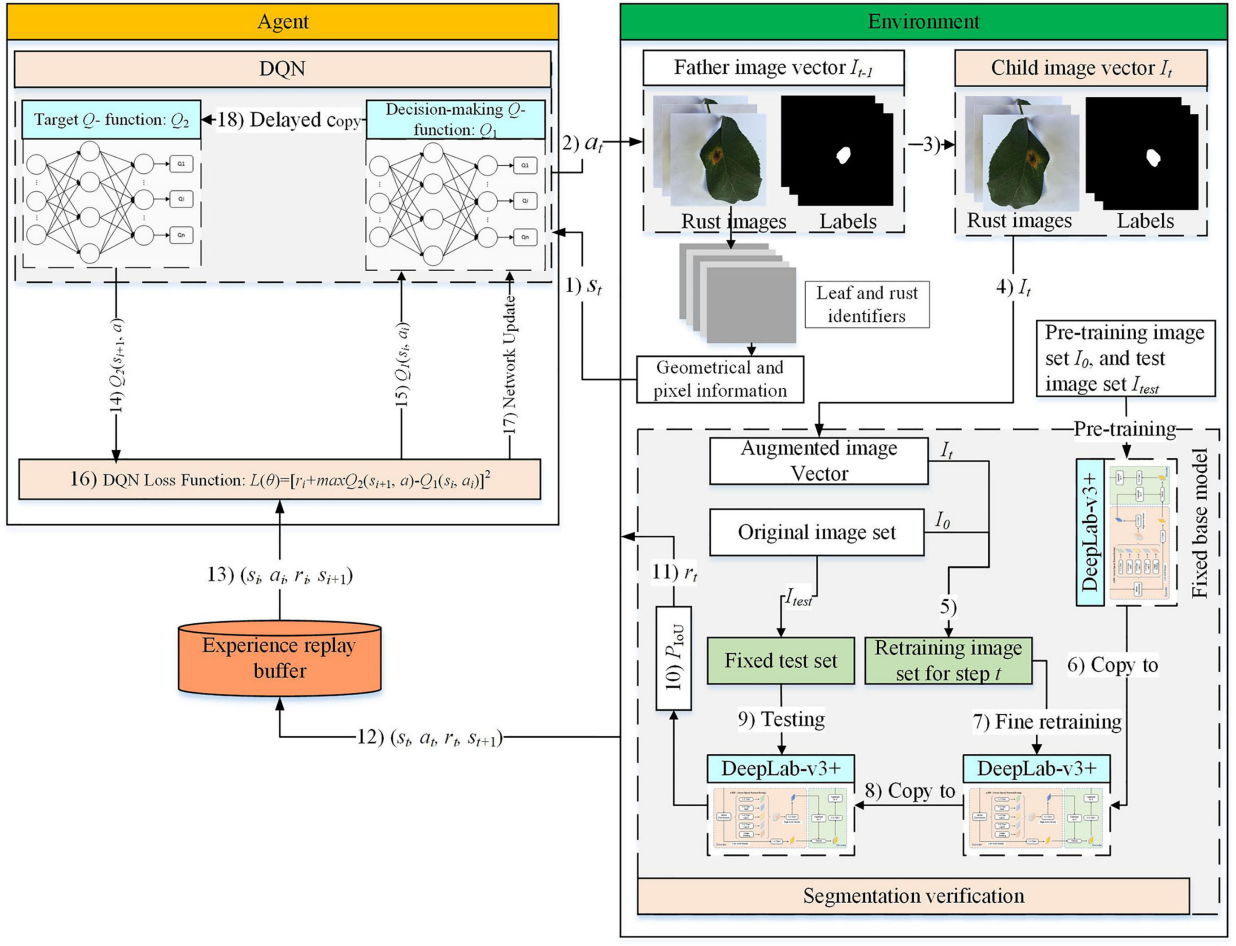
DRL-enabled adaptive image augmentation framework.

The DRL-enabled adaptive image augmentation framework is depicted in **Figure 1**. The DQN model acts as the Agent, and the image set is treated as the environment. The Agent and the Environment repeatedly interact through the signals: state $s_{t}$ , action $a_{t}$ , and reward $r_{t}$ . The state $s_{t}$ and the reward $r_{t}$ are output by the environment to the Agent while the action $a_{t}$ is determined by the Agent and executed in the environment. The interaction process consists of episodes, which in turn comprise multiple steps. The experience data are collected during the interaction process and used to train the Agent until the Agent can best match the augmentation methods and the images. In this specific scenario, the Agent can augment a given image appropriately so that the augmented image set can enable the segmentation model to output better performance.

**Figure 2:**
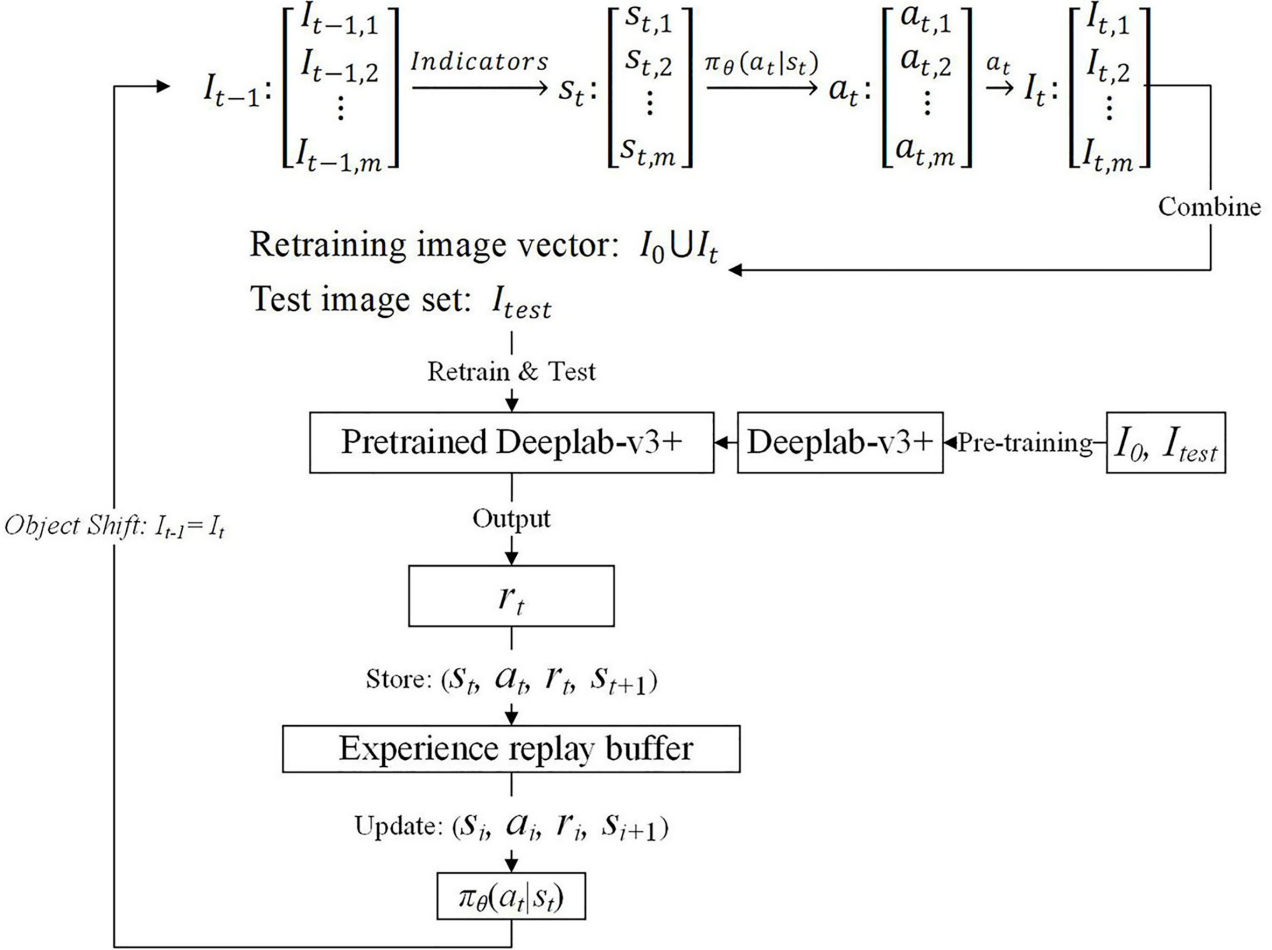
DRL-enabled adaptive image augmentation process.

The detailed interaction process is illustrated in **Figure 2**. A group of objects, e.g., images, states, and actions, are represented as a vector when the precedence relationship should be maintained; otherwise, the group of things is encapsulated with a set. In any round of interaction t , the geometric and pixel indicators are applied to extract the image features of the father image vector $I_{t-1}$ , which are then used to construct the state vector $s_{t}$ . After that, the action vector $a_{t}$ is determined based on the state vector $s_{t}$ and the Agent policy function $\pi_{\theta }(a_{t}|s_{t})$ . The actions in $a_{t}$ represent image augmentation methods selected individually for each image in $I_{t-1}$ . Therefore, $a_{t}$ will produce a child image vector $I_{t}$ after being executed. After that, the child image vector is combined with the pre-training image set $I_{0}$ to construct a retraining image set. Then, the retraining image set is used to retrain the pre-trained image segmentation model, DeepLab-v3+. Finally, the retrained model is tested on the test image set $I_{test}$ , and the testing results are used to generate the reward $r_{t}$ . At this moment, the data $\left(s_{t},a_{t},r_{t}\right)$ can be collected.

In the next round, the $I_{t}$ is used as the father image vector, and the above process is repeated so that the data $\left(s_{t+1},a_{t+1},r_{t+1}\right)$ can be collected. In addition, the data $\left(s_{t},a_{t},r_{t},s_{t+1}\right)$ need storing in the experience replay buffer for training the Agent policy function $\pi_{\theta }(a_{t}|s_{t})$ . After the process is repeated T times, an episode is said to be completed. To begin the next episode, reset t to 1, and restore the pre-training image set $I_{0}$ as the father image vector. The number of episodes, L , is another hyperparameter like the number of steps T within an episode, which means a total of L by T steps should be executed.

The Agent policy function $\pi_{\theta }(a_{t}|s_{t})$ evolves during the above interaction process. A number of S samples are extracted from the experience replay buffer and applied to update the parameter θ of $\pi_{\theta }(a_{t}|s_{t})$ . The hyperparameters, e.g., L , T , and S need adjusting and $\pi_{\theta }(a_{t}|s_{t})$ need updating till the performance is satisfied.

### 2.1 Image set and image vector

The original image set is divided into two subsets. Twenty percent of the images are sampled randomly from the original image set, forming the test image set $I_{test}$ that is used to test the DeepLab-v3+ model. The remaining 80% of images are collected by a subset denoted as $I_{0}$ , which is called the pre-training image set. Let $I_{0}=\left\{\;I_{0,1},I_{0,2},\ldots ,\;I_{0,m}\right\}=\left\{\left(x_{1}^{0},y_{1}^{0}\right),\left(x_{2}^{0},y_{2}^{0}\right),\ldots ,\;\left(x_{m}^{0},y_{m}^{0}\right)\right\}$ , where $x_{i}^{0}$ and $y_{i}^{0}$ are the *i*th image and its corresponding label image, and *m* is the total number of samples in the image set. Through the image augmentation procedure, an image in $I_{t-1}\left(t=1\ldots T\right)$ is applied to an image augmentation method to produce an augmented image, and all the augmented images make up the augmented image set $I_{t}=\left\{\;I_{t,1},I_{t,2},\ldots ,\;I_{t,m}\right\}=\left\{\;\left(x_{1}^{t},y_{1}^{t}\right),\left(x_{2}^{t},y_{2}^{t}\right),\ldots ,\;\left(x_{m}^{t},y_{m}^{t}\right)\right\}$ .

During the DQN augmentation process, the image sets are represented as vectors. In an image vector, the images are queued in a line, each occupying a fixed and unique position. At the first step of an episode, i.e., t=1 , $I_{0}$ is used as the father image vector denoted as $I_{t-1}$ . Then the images in $I_{t-1}$ are augmented to produce the child image vector denoted as $I_{t}$ . The image vectors are used instead of image sets because the corresponding relationship between $I_{t-1}$ and $I_{t}$ should be maintained. In other words, the first image in $I_{t}$ is produced from the first image in $I_{t-1}$ and so forth. It is noted that the images in $I_{t-1}$ are applied to image augmentation methods independently.

The pre-training image set $I_{0}$ alone is used to pre-train the DeepLab-v3+ model. In contrast, $I_{0}$ is combined with the augmented image set $I_{t}$ to retrain the pre-trained DeepLab-v3+ model to verify the effect of $I_{t}$ . In other words, the $I_{0}$ and $I_{test}$ are used to pre-train and test the semantic segmentation model DeepLab-v3+. The pre-trained DeepLab-v3+ model is retrained and tested by $I_{0}U^{}I_{t}$ and $I_{test}$ to see the influence of the augmented image set $I_{t}$ on the pre-trained model.

In the next step, the newly produced image vector $I_{t}$ instead of $I_{t-1}$ is used as the father image vector to produce its child image vector $I_{t+1}$ . Then, $I_{t+1}$ is united with $I_{0}$ to construct another retraining image set to test the augmentation effect of $I_{t+1}$ based on the pre-trained DeepLab-v3+ model. To sum up, the newly produced child image vector is used as the father image vector in the next step until the episode ends. However, to begin a new episode, the pre-training image set $I_{0}$ is used as the father image vector again, and the image vectors produced in the last episode are discarded. It is noted that the pre-trained DeepLab-v3+ model is restored in every retraining process and is used as a base model to observe the effect of the augmentation methods on the augmented image sets.

### 2.2 MDP model for DRL

The DRL-based optimization features a Markov decision process (MDP) (Han et al., 2021). The Agent selects an action from the candidate’s actions based on the current state of the environment. The execution of the action will introduce a state change to the environment which in turn generates a reward to the Agent. The Agent decides (i.e., selects an action) based on the current state only, not depending on the previous states. This design contributes to simplifying the Agent policy function but requires sophisticated state representation. The reward guides the evolution of the policy function. Therefore, maximizing cumulative compensation should correspond to the best selection policy of augmentation methods for any given image set. Although the single-step reward can be positive (a prize), negative (a penalty), or zero, the Agent should tolerate the short-term penalty while pursuing the maximum cumulative reward. The actions are candidate image augmentation methods that have been proven to be effective in certain circumstances. The best state-action match, however, is still unknown, leaving optimization space for DRL. Therefore, the state, action, and reward design will significantly influence DRL’s optimization quality (Ladosz et al., 2022).

#### 2.2.1 State

An amount of information is extracted from the image vector to describe the state of the environment. In this study, each image’s geometrical information and pixel information comprise a state for a given image vector. At first, one segmentation model, called LeafIdentifier, is trained to separate a leaf from its background. Furthermore, the other segmentation model, called RustIdentifier, is trained to separate the rust from a leaf. The LeafIdentifier and the RustIdentifier models are developed based on the DeepLab-v3+ model but prepared with different datasets. The image set $I_{0}$ with the leaf label is used to train the LeafIdentifier model, while the image set $I_{0}$ with the rust label is used to train the RustIdentifier model.

After that, the centroid and area of the leaf and the rust can be calculated. In addition, the pixel values can be averaged according to the RGB color channels for the leaf and the rust, respectively. Therefore, a state element that describes the *i*th image is:


$$s_{t,i}=\left\{x_{l,i},y_{l,i},A_{l,i},R_{l,i},G_{l,i},B_{l,i},x_{r,i},y_{r,i},A_{r,i},R_{r,i},G_{r,i},B_{r,i}\right\}$$

where, $x_{l,i}$ and $y_{l,i}$ are the centroid coordinates of a leaf, $A_{l,i}$ is the area of a leaf, and $R_{l,i}$ , $G_{l,i}$ , and $B_{l,i}$ are the average pixel values of a leaf, corresponding to the RGB color channels, respectively; $x_{r,i}$ , $y_{r,i}$ , $A_{r,i}$ , $R_{r,i}$ , $G_{r,i}$ , and $B_{r,i}$ are the corresponding elements for the rusts on the leaf.

Therefore, the state vector has the same number of elements as the father image vector, and their elements have a one-to-one corresponding relationship.

#### 2.2.2 Action

Eight kinds of image augmentation methods are selected as actions, as shown in **Table 1**. The *original image* operation does not change the image. The *vertical flip* operation makes an image flip vertically, while the *horizontal flip* operation makes an image flip horizontally. However, the *vertical and horizontal flip* operations apply the two operations together to a single image. The *clockwise rotation* operation causes an image to rotate 30° clockwise around the center point. The *affine transformation* is a type of geometric transformation that preserves collinearity and the ratios of distances between points on a line. The *crop* operation is to crop the original image and then resize it to the original size. When applying the noise-adding operation, random white Gaussian noise will be added to a given image. Each image augmentation method is assigned a unique number, i.e., 0, 1, 2,…7. In this study, $a_{i}\left(i=0\ldots 7\right)$ is used to represent the eight candidates’ actions, and $a_{t}\left(t=1\ldots T\right)$ is used to indicate the action vector consisting of actions selected independently for each image in the decision step t . Therefore, the different elements of $a_{t}$ possible correspond to the same $a_{i}$ .

**Table 1:** Action definition.

a_i_	Actions	Examples	Description
0	Original image	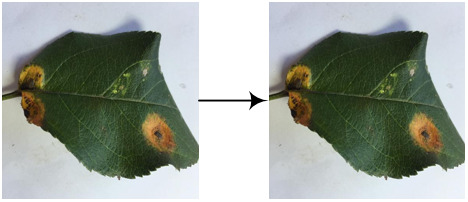	The resultant image is the same as the original one.
1	Vertical flip	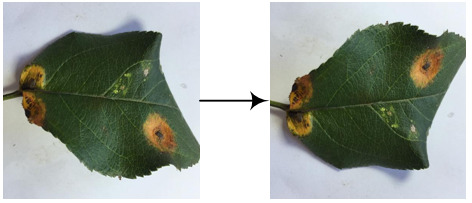	The resultant image mirrors the original one along the horizontal center line.
2	Horizontal flip	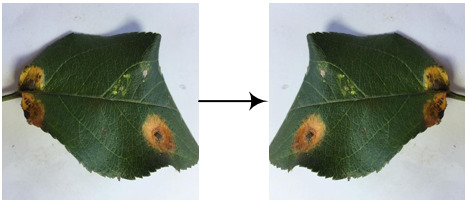	The resultant image mirrors the original one along the vertical center line.
3	Vertical and horizontal flip	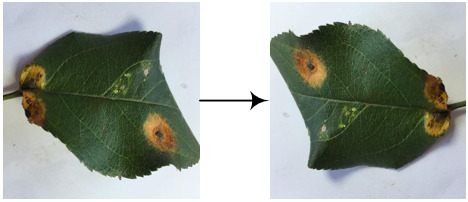	The original image is flipped vertically and horizontally to produce the resultant image.
4	Clockwise rotation	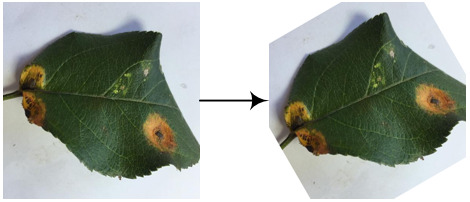	The original image is rotated 30° clockwise around the center point to produce the resultant image.
5	Affine transformation	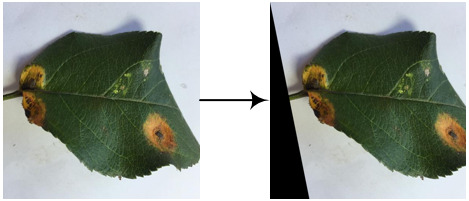	The original image is transformed with the matrix [[1, 0.2, 0], [0, 1, 0]] to produce the resultant image.
6	Crop	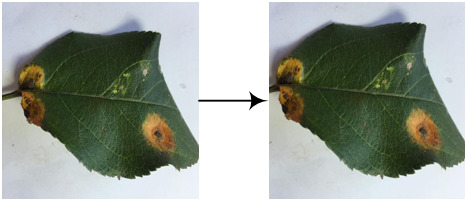	The first 25 rows and 25 columns of pixels of the original image are trimmed and then the image is resized to 512 × 512 pixels to produce the resultant image.
7	Noise-adding	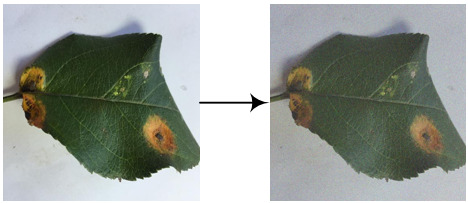	Some random white Gaussian noise is added to the original image to produce the resultant image.

#### 2.2.3 Reward

The reward is a numerical evaluation of an action selected by the Agent:


(1) $$r_{t}=100\left(d_{t}-d_{t-1}\right)$$

where, $d_{t}$ refers to the Dice ratio, defined as follows:


(2) $$d_{t}=\frac{2}{\left|I_{test}\right|}\sum_{(x_{j},y_{j})\in I_{test}}P_{IoU}$$

where, $\left|I_{test}\right|$ is the number of elements in the test image set $I_{test}$ , and $P_{\text{IoU}}\in \left[0,1\right]$ represents the segmentation effect of the retrained DeepLab-v3+ model on an image of $I_{test}$ :


(3) $$P_{\text{IoU}}=\frac{\left|{\hat{y}}_{j}\cap y_{j}\right|}{\left|{\hat{y}}_{j}\cup y_{j}\right|},\;y_{j}\in \left(x_{j},y_{j}\right)\in I_{test}$$

where, ${\hat{y}}_{j}$ is the predicted label image output by the retrained DeepLab-v3+ model, and $y_{j}$ is the expected label image, both for the image $x_{j}$ in the test image set $I_{test}$ ; $\left|{\hat{y}}_{j}\cap^{}y_{j}\right|$ and $\left|{\hat{y}}_{j}\cup^{}y_{j}\right|$ are the intersection and union area of the predicted and expected label images, respectively:


(4) $${\hat{y}}_{j}=f\left(x_{j};\theta_{I_{0}\cup I_{t}}\right),\;x_{j}\in \left(x_{j},y_{j}\right)\in I_{test}$$

where f denotes the retrained DeepLab-v3+ model, and $\theta_{I_{0}U^{}I_{t}}$ denotes the parameters updated by the retraining image set $I_{0}U^{}I_{t}$ .

To sum up, $d_{i}^{t}$ indicates the overall influence of the selected augmentation methods, $a_{t}$ , for a given image vector $I_{t}$ . As every $I_{t}$ is used to retrain the same pre-trained Deeplab-v3+ model, and the retrained DeepLab-v3+ model is tested on the same test image set $I_{test}$ , $d_{i}^{t}$ can be used for augmentation effect comparison and reward calculation.

### 2.3 Semantic segmentation model

A semantic segmentation model is integrated into the framework to evaluate the image augmentation effect. Based on the evaluation results, rewards can be produced, and feedback can be provided to the DQN model, which adjusts the Agent policy function accordingly.

#### 2.3.1 Model selection

At present, plant disease segmentation methods based on deep learning mainly include semantic segmentation and instance segmentation. Instance segmentation is more potent as it can distinguish different objects, while semantic segmentation can only determine things from the background. However, the semantic segmentation method is a better choice for this study, as it can meet the verification requirements, is simple and requires less computing resource consumption.

Deep learning-based semantic segmentation methods can improve accuracy and efficiency significantly compared with traditional methods. Currently, commonly used deep learning semantic segmentation models include FCN (Long et al., 2015), U-Net (Ronneberger et al., 2015), SegNet (Badrinarayanan et al., 2017), and DeepLab (Chen et al., 2014). The specific analysis is shown in **Table 2** (Chen et al., 2017). It can be seen that the DeepLab-v3+ model (Chen et al., 2018) has the highest accuracy and the best application effect. Therefore, the DeepLab-v3+ model is used in this study.

**Table 2:** Performance comparison of deep learning-based semantic segmentation models.

Proposed time	Network model	Segmentation accuracy	Training time	Algorithm Features
2014	FCN	C	B	Based on the CNN network, it introduces a deconvolution layer.
2014	DeepLab-v1	B	C	It combines dilated convolutions with DCNN networks and optimizes with fully connected conditional random fields.
2015	U-Net	B	–	It is completely symmetrical and the decoder is added with convolution and deepening.
2016	DeepLab-v2	B	C	It uses dilated convolutional layers instead of up-sampling and uses multi-scale spatial pyramid pooling.
2017	SegNet	C	C	It utilizes the encoder-decoder network structure and recovers the image size by up-sampling.
2018	DeepLab-v3+	A	C	It uses an encoder-decoder network structure to improve the segmentation of object edges and introduces dilated convolutions.

A. Very Good, B. Good, C. Fair.

The DeepLab-v3+ model can convert an image into a prediction highlighting diseased areas from the background (Tian et al., 2019). In the rust detection application, each pixel in the apple rust leaf image is assigned to one of the mutually exclusive classes: disease spots VS background, to complete the segmentation of disease spots from the background (Kuang and Wu, 2019).

#### 2.3.2 Deeplab-v3+ model

**Figure 3:**
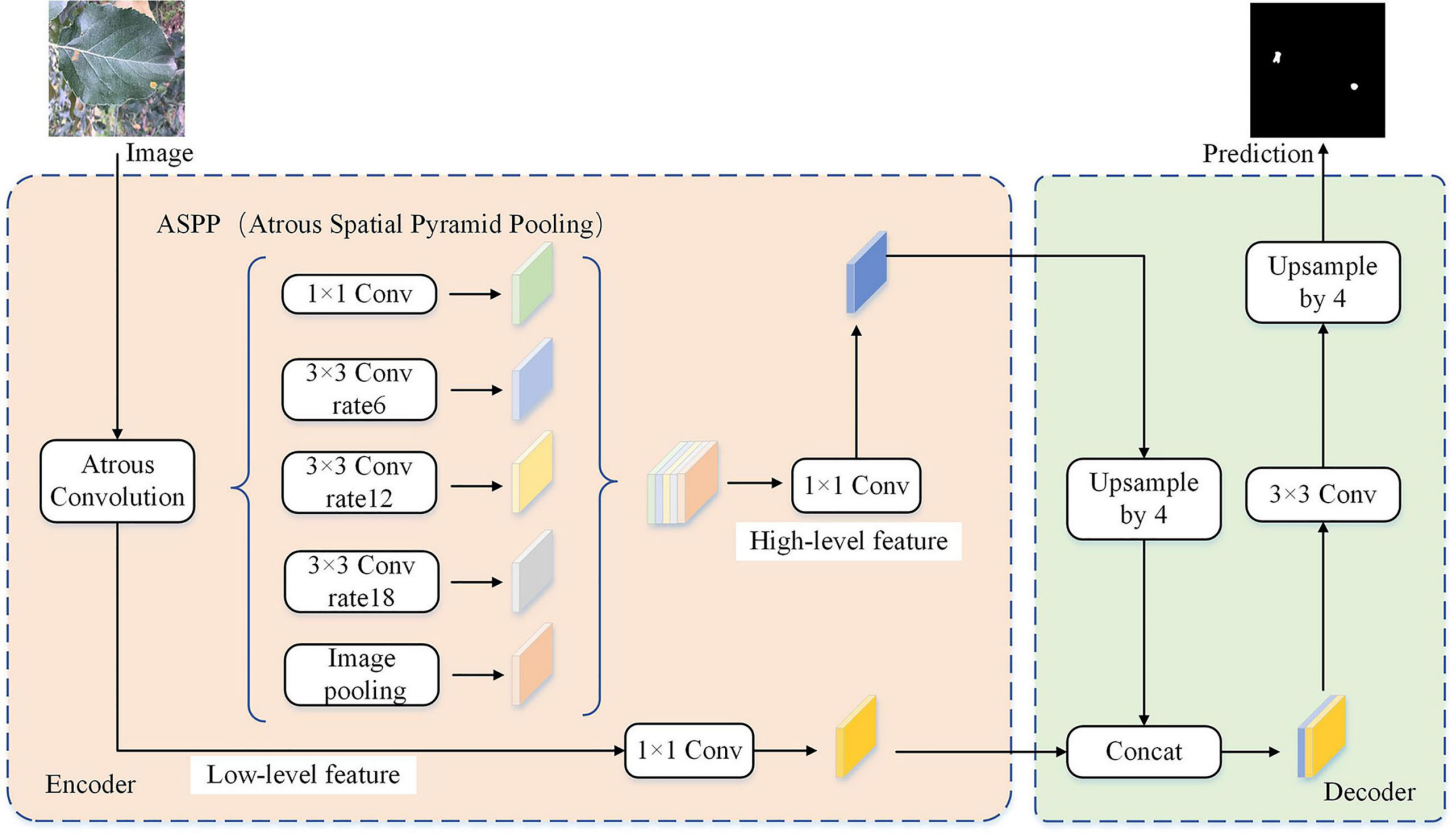
The network structure of the DeepLab-v3+ model.

As shown in **Figure 3**, the DeepLab-v3+ model adds a simple and effective decoder layer to the DeepLab-v3 model to refine the segmentation results. Furthermore, in the Encoder part, the Atrous Spatial Pyramid Pooling (ASPP) module is constructed using Atrous convolution and the Spatial Pyramid Pooling module (SPP). Atrous convolution is the process of adding spaces between convolution kernel elements to expand the convolution kernel. The SPP performs pooling operations at different resolution levels to capture rich contextual information. Consequently, five different outputs are obtained through the five distinct processes of ASPP to produce a high-level feature, and the Atrous convolution outputs a low-level component. In the Decoder part, the high-level feature is first up-sampled by 4 and then connected with the low-level quality. The concatenation passes through 3 × 3 convolutions and is then up-sampled by 4 to give the predicted label image.

#### 2.3.3 Model evaluation

To evaluate the segmentation effect of the DeepLab-v3+ model from multiple perspectives, the confusion matrix is calculated (Chen and Zhu, 2019), as shown in **Table 3**.

• *K*_TP_ is the true positive, indicating the number of disease spot pixels that are correctly classified into the disease spot region.
• *K*_FP_ is the false positive, indicating the number of background pixels that are wrongly classified into the disease spot region.
• *K*_TN_ is the true negative, indicating the number of background pixels that are correctly classified into the background region.
• *K*_FN_ is the false negative, indicating the number of disease spot pixels wrongly classified into the background region.

**Table 3:** Confusion matrix of disease spot detection.

Pixel point classification area		Expected class	
		Disease spot	Background
Predicted class	Disease spot	$K_{TP}$	$K_{FP}$
	Background	$K_{FN}$	$K_{TN}$

After that, five performance indexes are defined based on $K_{\text{TP}}$ , $K_{\text{FP}}$ , $K_{\text{TN}}$ , and $K_{\text{TN}}$ (Wang et al., 2020).


(5) $$P_{A}=\frac{K_{\text{TP}}+K_{\text{TN}}}{K_{\text{TP}}+K_{\text{TN}}+K_{\text{FP}}+K_{\text{FN}}}$$

where, $P_{A}\in \left[0,1\right]$ tells how many pixels are correctly classified relative to the total number of pixels.


(6) $$P_{MPA}=\frac{1}{2}\left(\frac{K_{\text{TP}}}{K_{\text{TP}}+K_{\text{FP}}}+\frac{K_{\text{TN}}}{K_{\text{TN}}+K_{\text{FN}}}\right)$$

where, $P_{\text{MPA}}\in \left[0,1\right]$ averages correctly classified disease spot pixels and background pixels relative to the predicted total disease spot pixels and the total background pixels, respectively.


(7) $$P_{CPA}=\frac{K_{\text{TP}}}{K_{\text{TP}}+K_{\text{FP}}}$$

where, $P_{CPA}\in \left[0,1\right]$ tells how many disease spot pixels are correctly classified relative to the predicted total disease spot pixels.


(8) $$P_{IoU}=\frac{K_{\text{TP}}}{K_{\text{TP}}+K_{\text{FN}}+K_{\text{FP}}}$$

where, $P_{IoU}\in \left[0,1\right]$ tells how many disease spot pixels are correctly classified relative to the union of the predicted and expected disease spot pixels.


(9) $$P_{\text{MIoU}}=\frac{1}{2}\left(\frac{K_{\text{TP}}}{K_{\text{TP}}+K_{\text{FN}}+K_{\text{FP}}}+\frac{K_{\text{TN}}}{K_{\text{TN}}+K_{\text{FP}}+K_{\text{FN}}}\right)$$

where, $P_{\text{MIoU}}\in \left[0,1\right]$ averages correctly classified disease spot pixels and background pixels relative to the union of the predicted and expected disease spot pixels and the union of the predicted and expected background pixels, respectively.

### 2.4 Model training

According to the MDP mentioned above and semantic segmentation models, the main training steps are summarized as follows:

• Preprocessing: Producing leaf labels and rust labels for the original image set and dividing it into the pre-training image set $I_{0}$ and the test image set $I_{test}$ ; pre-training the DeepLab-v3+ model with $I_{0}$ , $I_{test}$ , and the leaf labels to generate the LeafIdentifier; pre-training the DeepLab-v3+ model with $I_{0}$ , $I_{test}$ , and the rust labels to generate the RustIdentifier; selecting DQN as the specific DRL model, and initializing the decision-making Q -function $Q_{1}$ and the target Q -function $Q_{2}$ for DQN.
• Image augmentation: Taking the child image vector in step t−1 , i.e., $I_{t-1}$ , as the father image vector in step t ; using the LeafIdentifier, RustIdentifier, and the geometric and pixel indicators to process the images in $I_{t-1}$ , one by one, to generate the state vector $s_{t}$ , i.e., the processing result of one image contributes one element in $s_{t}$ ; using $Q_{1}$ to determine one action for each state element, generating the action vector $a_{t}$ , and one state element corresponds to one action element; executing the action elements in $a_{t}$ to the corresponding image elements in $I_{t-1}$ to produce the child image vector $I_{t}$ ; getting $s_{t+1}$ from $I_{t}$ .
• Verification: Constructing the retraining image set, the element of which is $I_{0}U^{}I_{t}$ that means $I_{0}$ plus $I_{t}$ gives a training image set; restoring the pre-trained DeepLab-v3+ model; fine retraining the model with $I_{0}U^{}I_{t}$ ; testing the retrained model against $I_{test}$ , storing the results, and calculating the reward $r_{t}$ ; storing $\left(s_{t},\;a_{t},\;r_{t},s_{t+1}\right)$ into the experience replay buffer.
• DQN network updating: Sampling a batch of data, ( $s_{i},\;a_{i},\;r_{i},s_{i+1}$ ), from the experience replay buffer; calculating the loss function, L(θ) , with $Q_{1}$ , $Q_{2}$ , and the sampled data; updating $Q_{1}$ with $L\left(\theta \right)={\left[r_{i}+\underset{a}{\max}Q_{2}\left(s_{i+1},\;a\right)-Q_{1}\left(s_{i},\;a_{i}\right)\right]}^{2}$ and the backpropagation algorithm; copying the parameters of $Q_{1}$ to $Q_{2}$ every C steps to update $Q_{2}$ . $Q_{2}$ is updated C times slower than $Q_{1}$ for improving stability.
• Starting the next step or a new episode: The above steps except preprocessing are repeated for every step of an episode until the episode ends. To start a new episode, the pre-training image set $I_{0}$ is restored as the father image vector for the first step of the episode, and the above steps except preprocessing are repeated until the episode ends.

In summary, the specific DRL algorithm, DQN, is used in this study to organize an adaptive image augmentation scheme. The DQN is assisted with the geometric and pixel indicators for state extraction, the DeepLab-v3+ model for verifying the augmented images and generating the reward, and the image augmentation methods as actions. The image and its accompanying label image are processed in the same way by the selected image augmentation method. The DeepLab-v3+ model is pre-trained once and restored for every retraining operation. DQN parameters keep updating through all the steps and episodes, i.e., they are not reset or restored from a previous step or episode.

## 3 Experimental results and discussion

### 3.1 Data sources and image preprocessing

**Figure 4:**
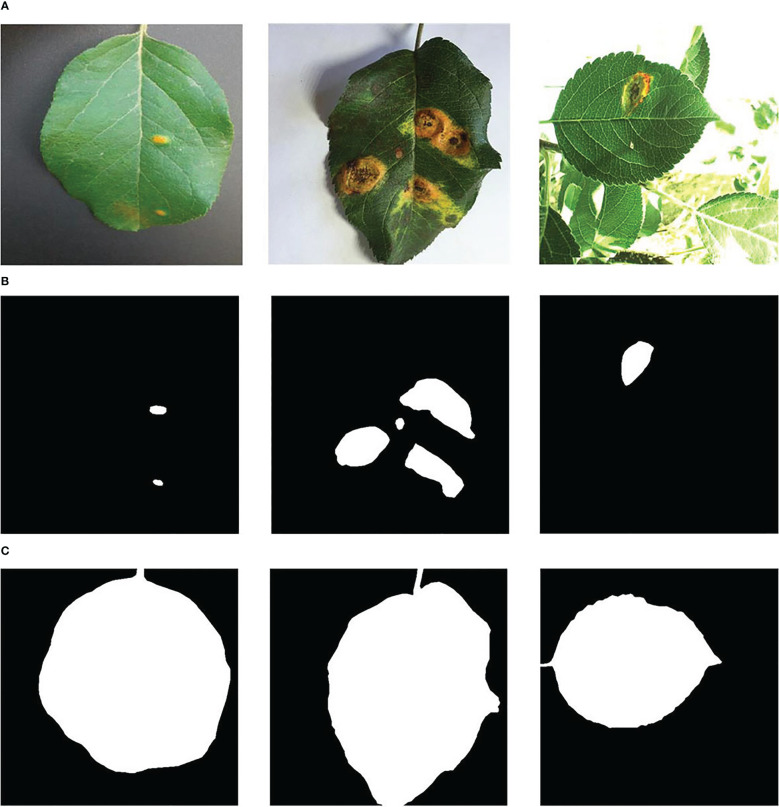
Samples of **(A)** the apple rust images, **(B)** the rust labels, and **(C)** the leaf labels.

The experimental data comes from the open-source apple leaf disease image dataset on the Baidu AI Studio Development platform, with a resolution of 512 × 512 pixels. Among them, there are 438 images of apple leaf rust, including images collected in various environments, all of which are used in this study. Some representative images are shown in **Figure 4A**. The EIseg software (Xian et al., 2016) uses the latest deep learning algorithms and models to greatly reduce annotation effort. Therefore, it is used to mark the image, distinguishing the disease spot areas and the whole leaf from the background, to produce labels, as shown in **Figures 4B, C**. The label images have the same resolution as the original images.

The image set was divided according to the ratio of 8:2, and the image and its label image would not separate during division. As a result, there were 350 images in the pre-training image set $I_{0}$ , and 88 images in the test image set $I_{test}$ , respectively.

### 3.2 DeepLab-v3+ model pre-training

The training hardware platform consisted of a Platinum 8358P CPU, a GTX 3090 GPU, and 24 GB of running memory. The software was built with the deep learning framework Pytorch. The testing results indicated that the DeepLab-v3+ model could process about 379 sets of images per second. During training, it took about 4 s to complete each epoch. As DeepLab-v3+ was set to 1,000 epochs in our experiment, it took about 4,000 s in total to complete the pre-training of the DeepLab-v3+ model.

**Figure 5:**
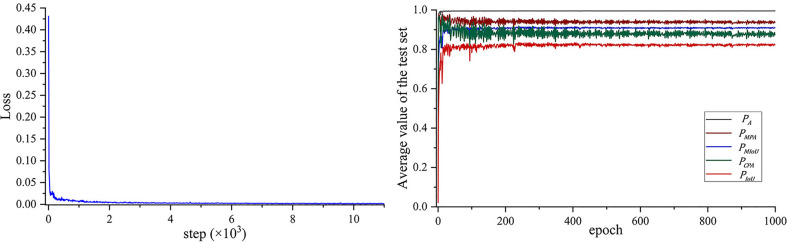
Training histories of **(A)** the loss and **(B)** the performance output on the test image set.

The loss curve and the five performance indexes are shown in **Figure 5**. The DeepLab-v3+ model converges after about 239 epochs, where the loss is about 3.42e−3. The average $P_{A},P_{MPA},\;P_{MIoU},P_{CPA},\text{ and }P_{IoU}$ are 0.9956, 0.9444, 0.9131, 0.8905, and 0.8307, respectively. In the verification stage, the pre-trained DeepLab-v3+ model is retrained with $I_{0}U^{}I_{t}$ in a fast-fine-tuning way. If the retrained DeepLab-v3+ model can output better performance, the augmented images $I_{t}$ are said to improve segmentation performance, which means the DRL model can select proper augmentation methods.

### 3.3 DQN model training

The hardware platform for DQN training consisted of a 24 vCPU AMD EPYC 7642 48-Core processor and a single NVIDIA GTX 3090 GPU with 24 GB of running memory. The DQN algorithm was developed with PyTorch and Python 3.8.10. For each training step of the proposed method, the image augmentation set could be generated in 25 s, and it took about 165 s to complete the parameter fine-tuning of the DeepLab-v3+ model and about 0.003 s to update the parameters of DQN. Therefore, it took about 3.16 min to complete each step and 9.48 min to complete one episode for the proposed method. As DQN was set to 300 episodes in our experiment, it took about 2,844 min in total.

**Figure 6:**
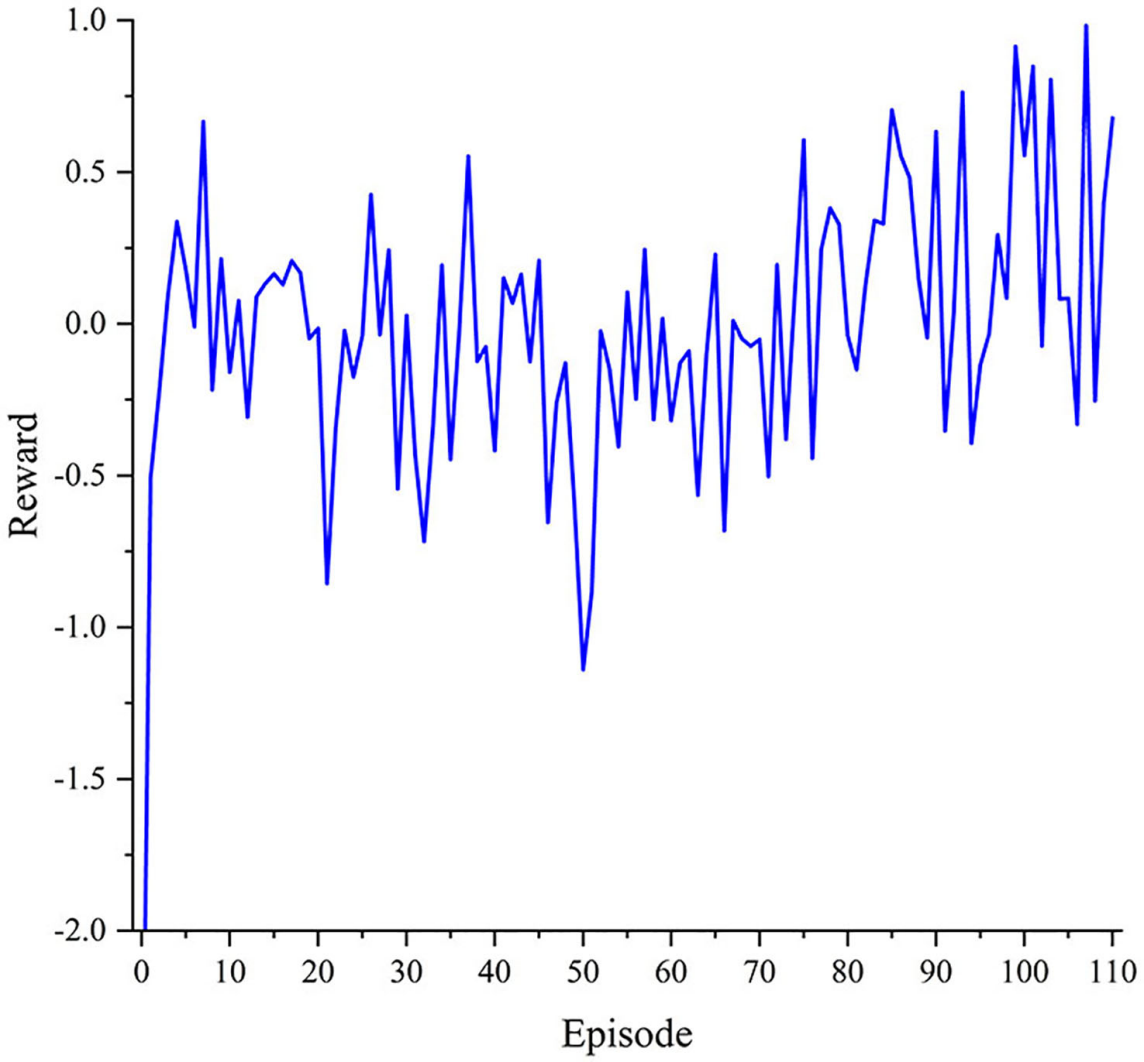
Training histories of the reward.

As shown in **Figure 6**, the reward is very small at the beginning, i.e., −2.975. As the training process progresses, the reward increases significantly and then fluctuates around zero. To sum up, the results show that the reward increases from −2.975 to 0.9826 during DQN training, achieving an improvement of nearly 3.958. That is to say, the effect of the DQN model on disease spot segmentation is greatly improved, which proves that the model can automatically learn how to adopt reasonable and most effective image augmentation methods according to the image features.

### 3.4 Performance comparison of the image augmentation methods

The DQN model was compared with every single method listed in **Table 1**, i.e., No. 0: *original image*; No. 1: *vertical flip*; No. 2: *horizontal flip*; No. 3: *vertical and horizontal flip*; No. 4: *clockwise rotation*; No. 5: *affine transformation*; No. 6: *crop*; and No. 7: *noise adding*. For the *i*th (i=0…7) image augmentation method, the images in $I_{0}$ were augmented by the same augmentation method to produce an augmented image set. Then $I_{0}$ was combined with the augmented image set to construct a retraining image set. The retraining image set was used to retrain the pre-trained DeepLab-v3+ model, and the retrained model was tested on the $I_{test}$ . This way, a separate set of performance indexes, e.g., $P_{IoU}$ and $P_{CPA\;},\;$ were produced for each image augmentation method for comparison.

**Figure 7:**
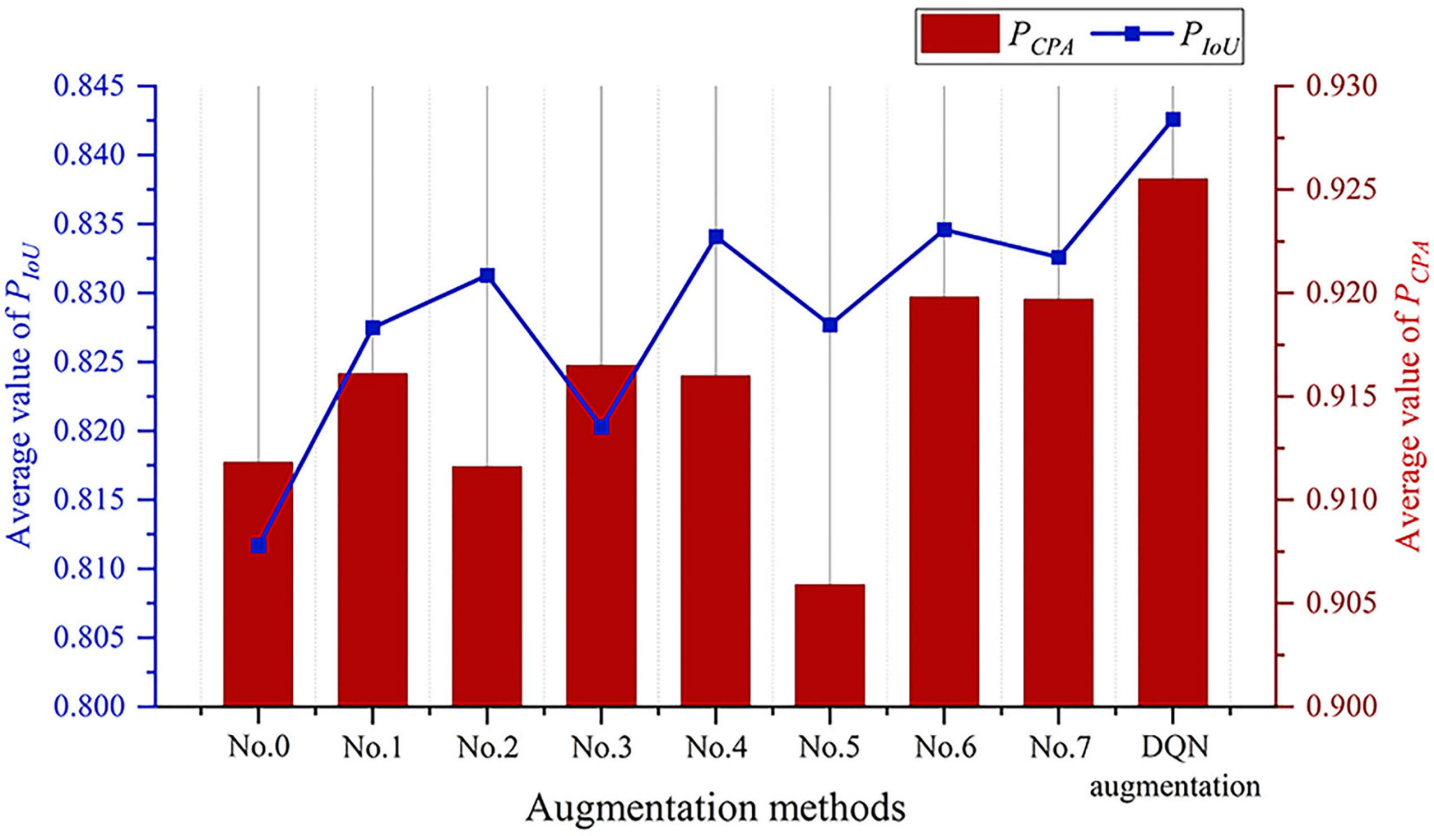
Augmentation effect of different methods.

**Figure 7** shows the augmentation effect of different methods. The *original image* augmentation method achieves an average $P_{IoU}$ value of 0.8117, which is the lowest. The *affine transformation* augmentation method achieves an average $P_{CPA}$ value of 0.9059, which is also the lowest. In contrast, the DQN augmentation method achieves the best performance, with $P_{IoU}$ value of 0.8426 and $P_{CPA}$ value of 0.9255. Therefore, this experimental result confirms the effectiveness of the DQN model in adaptively selecting the augmentation methods according to the image features. The testing results showed that the DQN model could generate 12 augmentation image sets (with labels) per second, and the performance was maximum.

### 3.5 Performance comparison of the semantic segmentation models

The DeepLab-v3+ model (denoted as DQN-DeepLab-v3+) was compared with the FCN and SegNet models. Firstly, the DQN-DeepLab-v3+, FCN, and SegNet models were pre-trained with $I_{0}$ and $I_{test}$ , respectively. Secondly, let the proposed DQN model output an augmentation image set. Thirdly, a retraining image set was constructed with $I_{0}$ and the augmented image set, and then the retraining image set was used to retrain the DQN-DeepLab-v3+, FCN, and SegNet models, respectively. Finally, the retrained DQN-DeepLab-v3+, FCN, and SegNet models were respectively tested on $I_{test}$ to get a separate set of average performance indexes for comparison.

DeepLab-v3+ with random augmentation (denoted as RanAug-DeepLab-v3+) was also constructed for comparison. RanAug-DeepLab-v3+ was pre-trained, retrained, and tested following the same procedure as the DQN-DeepLab-v3+, FCN, and SegNet models. The only difference was that a random augmented image set was used instead of the expanded image set output by the DQN model. Furthermore, the test results of the pre-trained DeepLab-v3+ model were used as the baseline, as any augmented images did not retrain it.

**Figure 8:**
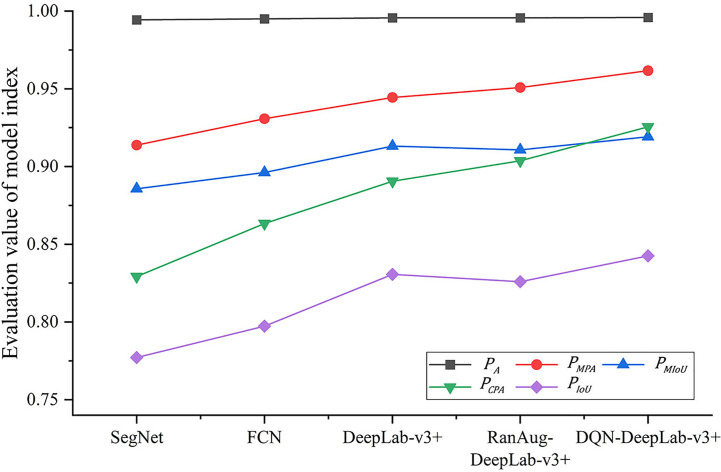
Segmentation effect of different models.

As shown in **Figure 8**, the proposed DQN-DeepLab-v3+ model achieves the best performance on all the indexes. $P_{A},\;P_{MPA},\;P_{MIoU},P_{CPA},\text{ and }P_{IoU}$ reaches 0.9959, 0.9617, 0.9192, 0.9255, and 0.8426, respectively, which are up to 0.2%, 3.7%, 3.9%, 7.3%, and 7.6% higher than other methods. In contrast, the SegNet achieves the worst performance, mainly by focusing on optimizing memory usage. The version of the FCN model is also relatively low due to the limited size of the perceptual area, easy loss of edge information, and low computational efficiency. These results confirm that the DQN-DeepLab-v3+ model is superior to the FCN and SegNet models. On the other hand, some performance indicators of RanAug-DeepLab-v3+ are lower than those of DeepLab-v3+, indicating that the random augmentation tends to harm the segmentation performance. In contrast, the DQN-DeepLab-v3+ model surpasses DeepLab-v3+, showing adaptive augmentation can improve segmentation performance.

## 4 Conclusion

Deep learning-based automated optical inspection can benefit from image augmentation, which enlarges the image quantity for training and testing. However, one significant challenge is that any single image augmentation method cannot achieve consistent performance over all the images. To address this issue, a DRL-enabled adaptive image augmentation framework is proposed in this paper. The specific DRL algorithm, DQN, is used in this study to organize an adaptive image augmentation scheme. Given an image vector, segmentation models and key indicators are used to extract image features and generate the state vector; the Agent policy function determines the action vector based on the state vector; and the actions produce an augmented image vector. To evaluate the image augmentation effect, a raised image is used to fine-tune a pre-trained semantic segmentation model, DeepLab-v3+, and the resultant model is tested against a fixed test image set. Based on the evaluation results, the reward is constructed, and feedback is sent to the DQN model, which updates the Agent policy function accordingly. Through iterations, the Agent policy function is optimized. The proposed DRL-enabled adaptive image augmentation framework achieves better augmentation performance than any single image augmentation method and better segmentation performance than other semantic segmentation models. The experimental results confirm that the DRL-enabled adaptive image augmentation framework can adaptively select augmentation methods that best match the images and the semantic segmentation model.

Future work should consider more advanced image augmentation methods, segmentation targets, and a more flexible and efficient DRL framework to provide more effective detection schemes for complex AOI application scenarios.

## Data availability statement

Publicly available datasets were analyzed in this study. This data can be found here: https://aistudio.baidu.com/aistudio/datasetdetail/11591.

## Author contributions

SW, AK, YL, ZJ, HT, SA, MS and UB were responsible for question formulation, method, experimental design, and manuscript writing. YL, ZJ, HT, SA, MS and UB contributed to the issue investigation. HT contributed to the data analysis and AK funded the research. All authors listed have made a substantial, direct, and intellectual contribution to the work and approved it for publication.
